# Synergistic remediation of cadmium and BDE-209 co-contaminated soil using *Solanum nigrum* assisted by Arbuscular mycorrhizal fungi and citric acid

**DOI:** 10.3389/fmicb.2025.1624164

**Published:** 2025-07-10

**Authors:** Hanhao Li, Rujun Zhou, Danyu Li, Xun Wen Chen, Cehui Mo, Hui Li

**Affiliations:** ^1^MOE Key Laboratory of Tumor Molecular Biology, College of Life Science and Technology, Jinan University, Guangzhou, China; ^2^Department of Ecology, Guangdong Provincial Research Centre for Environment Pollution Control and Remediation Materials, College of Life Science and Technology, Jinan University, Guangzhou, China

**Keywords:** co-contaminated soil, debromination, heavy metals, polybrominated diphenyl ethers, phytoremediation, polyphenol oxidase activity

## Abstract

Co-contamination of cadmium (Cd) and polybrominated diphenyl ethers (PBDEs) in soil is common, posing serious ecological and health risks. Simultaneous remediation of both pollutants using plants is particularly challenging due to their contrasting environmental behaviors. The challenge is chelators can enhance Cd extraction by plants but Cd inhibits microbial activity, limiting PBDEs degradation. To tackle this, arbuscular mycorrhizal (AM) fungi show promising potential as they produce extensive hyphae networks capable of immobilizing Cd and enhancing rhizosphere microbial activity. However, the combined effects of AM fungi and chelators for the simultaneous remediation remain elusive. Here, using a pot experiment, *Solanum nigrum* was grown in Cd/BDE-209 co-contaminated soil under four treatments (control, citric acid, AM fungi, and combined) to assess remediation potential. we found that CA increased ethanol-extractable Cd in shoots by 2.81-fold while reducing shoot total Cd concentration by 19.91%. Additionally, CA enhanced BDE-209 accumulation by 40.75% but decreased biomass by 20.22%. AM fungi increased the proportion of residual Cd in shoots, which thereby reduced Cd toxicity to plants, and enhanced the proportion of acid-soluble Cd in soil, promoting Cd mobilization. However, these changes did not affect the remaining Cd or BDE-209 concentrations in the soil. The combination of AM fungi and CA reduced soil Cd concentration by 13.09% compared to the control and promoted BDE-209 accumulation in *S. nigrum* shoots, resulting in a 42.80% decrease in soil BDE-209 concentration. This reduction was attributed to enhanced soil polyphenol oxidase and urease activities, which accelerated BDE-209 debromination and dissipation. Our work shows the synergistic potential of AM fungi and CA in mitigating Cd and PBDEs co-contamination, offering a sustainable remediation strategy.

## 1 Introduction

As of 2023, the global accumulation of unrecovered electronic waste has exceeded 347 million tons, and the waste recycling rate stands at only 22.3% (Anderson and Lee, 2025). Improper handling and disposal of electronic waste have led to the release of additives from electronic products into the environment, particularly soil. Among these additives, polybrominated diphenyl ethers (PBDEs), used as flame retardants in electronic products, are highly toxic (Vishwakarma et al., 2023). For example, BDE-209, the predominant congener of PBDEs, accounts for approximately 80% of global PBDE consumption and may degrade into lower brominated congeners, further increasing environmental risks (Sun et al., 2024). Unfortunately, soils contaminated with PBDEs are often co-contaminated with heavy metals, such as cadmium (Cd), due to the complex nature of e-waste composition and the release of these metals during recycling processes (Wei et al., 2023). This co-contamination poses significant ecological and health risks because Cd is highly mobile, difficult to mineralize, and can be absorbed by plants, entering the food chain and ultimately threatening human health. The remediation of co-contaminated soils is challenging due to distinct environmental behaviors, adsorption competition, and conflicting remediation requirements between heavy metals and organic pollutants. For example, heavy metals bind via ion exchange while organics rely on hydrophobic interactions, hindering simultaneous remediation (Nie et al., 2024).

Phytoextraction, using hyperaccumulator plants to take up soil pollutants, has often been used to remediate single pollutants effectively (Wang et al., 2023; Liu et al., 2024). However, its application is often hindered in co-contaminated soils, where heavy metals and organic pollutants coexist, due to their divergent environmental behaviors. To overcome this limitation, the integration of phytoextraction with chelating agents or microorganisms has been proposed as a viable strategy. Chelating agents, such as low molecular weight organic acids (LMWOAs), can dissolve heavy metals from the soil matrix into the soil solution, thereby improving their uptake by hyperaccumulator plants (Zhang et al., 2020). In parallel, microorganisms contribute to the degradation of organic pollutants, such as PBDEs, through enzymatic processes, converting them into less toxic or non-toxic metabolites (Li et al., 2018a). The combined use of chelating agents and microorganisms leverages their complementary mechanisms: chelating agents mobilize heavy metals, while microorganisms degrade organic pollutants. This synergistic approach not only enhances the overall efficiency of pollutant removal but also mitigates the ecological and health risks associated with co-contaminated soils. However, the mobilization of heavy metals by chelating agents may negatively impact microbial activity, potentially compromising the degradation of organic pollutants and overall remediation efficiency (Yang et al., 2025).

Arbuscular mycorrhizal (AM) fungi that form symbiotic relationships with more than 80% of terrestrial plants, can increase the phytoextraction efficiency of heavy metals or organics (Lenoir et al., 2016). By improving nutrient uptake and stress tolerance, AM fungi promote plant survival and growth in contaminated soils (Li H. H. et al., 2024). Furthermore, their extensive hyphal networks create ecological niches for soil microorganisms, potentially mitigating the adverse effects of chelating agents on microbial communities (Vieira et al., 2025). However, the effectiveness of AM fungi in addressing mineral-bound heavy metal contamination is limited, as they are often unable to mobilize heavy metals tightly bound within soil particles (Chen et al., 2022). To overcome this limitation, chelating agents such as citric acid can be employed to release these metals by forming stable complexes, thereby increasing their solubility and bioavailability for plant uptake. By combining the ability of AM fungi to support microbial communities and the metal-mobilizing capacity of citric acid, their synergistic effects can be harnessed to optimize the remediation of soils co-contaminated with heavy metals and PBDEs.

The efficiency of phytoextraction is highly dependent on plant species (Li M. et al., 2024). We selected *S. nigrum* as our model hyperaccumulator based on its documented capabilities: (1) It is the most prevalent Cd hyperaccumulator worldwide, exhibiting superior environmental adaptability and biomass production in Cd-contaminated soils compared to alternatives like *A. hypochondriacus* and *C. argentea* (Huang et al., 2020); (2) It demonstrates unique capacity for polybrominated diphenyl ether (PBDE) absorption from contaminated sewage sludge (Yao et al., 2021); (3) AM fungal inoculation (achieving 83.2% colonization with *RI* strains) can enhance Cd accumulation in its shoots by 69–249% (Liu et al., 2015; Li et al., 2018a). Previous studies have systematically investigated the remediation of Cd- and Pb-contaminated soils using *S. nigrum* combined with CA or microorganisms, focusing on plant growth, metal uptake, antioxidative enzyme activities, and soil enzyme activities (Gao et al., 2010b). Additionally, the role of AM fungi in enhancing Cd uptake by *S. nigrum* has been explored (Liu et al., 2015; Jiang et al., 2016). However, only Yang et al. (2011) have examined the use of *S. nigrum* for the phytoremediation of soils co-contaminated with Cd and polycyclic aromatic hydrocarbons (PAHs) using chemicals such as EDTA, cysteine, and salicylic acid. To date, few studies have addressed the simultaneous uptake of Cd and BDE-209 by *S. nigrum* combined with AM fungi and CA.

This study aims to evaluate the potential of *S. nigrum* combined with AM fungi (*Rhizophagus intraradices, Ri*) and CA for remediating soils co-contaminated with Cd and BDE-209. The specific objectives are to (1) assess the role of AM fungi and CA in enhancing plant growth and phytoextraction efficiency, (2) investigate the debromination of BDE-209 within the soil-plant system, and (3) identify effective remediation strategies for soils co-contaminated with Cd and BDE-209.

## 2 Materials and methods

### 2.1 Chemicals

Cadmium chloride (CdCl_2_⋅5H_2_O) and a standard Cd sample were obtained from Tianjin Kemiou Chemical Reagent Co., (China) and the Test Center of Steel Material (China), respectively. BDE-209 (C_12_Br_10_O) and HPLC-grade organic solvents (n-hexane, dichloromethane, methylbenzene, acetone) were supplied by Sigma-Aldrich (USA). A PBDEs standard solution (27 congeners, including BDE-3, –7, –15, –17, –28, –47, –49, –66, –71, –77, –85, –99, –100, –119, –126, –138, –153, –154, –156, –183, –184, –191, –196, –197, –206, –207, –209) and surrogate standards (MBDE-47, MBDE-209, FBDE-4003S) were purchased from Wellington Laboratories (Canada). Additional reagents (citric acid, anhydrous Na_2_SO_4_, alumina, silica gel) were procured from Guangzhou Chemical Reagent Factory (China). Anhydrous Na_2_SO_4_, silica gel, and alumina were activated at 150°C overnight. All experiments used distilled water.

### 2.2 Experimental design

Soil samples were collected from the 0–20 cm depth zone in paddy fields at South China Agricultural University, Guangzhou, China. The soil was characterized by 8.1% organic matter, 1.3 g kg^–1^ total nitrogen (N), 1.1 g kg^–1^ total phosphorus (P), and 0.61 g kg^–1^ total potassium (K), with a pH of 5.9. The background concentration of Cd was determined to be 0.18 mg kg^–1^, and no detectable levels of BDE-209 were observed.

The soil was air-dried for 2 weeks, sieved through a 2-mm nylon mesh, and autoclaved at 121°C for 2 h to remove indigenous microorganisms. After sterilization, Cd contamination was introduced by spiking with aqueous CdCl_2_, followed by 4-week incubation. The soil was then re-dried, homogenized, and re-sieved (2-mm mesh). BDE-209 in dichloromethane was added following Lu and Zhang (2014), and the spiked soil was dried in the dark until solvent evaporation. Final soil concentrations of Cd and BDE-209 were 14.8 mg kg^–1^ and 4.98 mg kg^–1^, respectively.

*S. nigrum* seedlings (South China Botanical Garden, China) were prepared by surface-sterilizing seeds with H_2_O_2_ (10 min), rinsing with deionized water (Wang G. et al., 2022), and germinating on moist filter paper in darkness. After 3 days, seedlings were transferred to basins with 20% Hoagland-Arnon solution as stock plants. For the pot trial, uniform 5-cm-tall seedlings were selected.

Four treatments combining AM fungi and CA were established as shown in Table 1. The AM fungi strain was obtained from Mycagro Lab (France). Each pot was filled with 1.6 kg of soil and inoculated with 20 g of mycorrhizal inoculum for AM fungi treatments. Five seedlings were planted per pot. Citric acid (5 mmol kg^–1^) was applied on days 20 and 27 for the chemical treatments. The selection of 5 mM citric acid concentration was based on previous studies demonstrating its effectiveness in alleviating heavy metal stress in plants from contaminated soils (Ijaz et al., 2022). Each treatment was performed in triplicate. To ensure sufficient nutrient availability, a 20% Hoagland-Arnon nutrient solution supplemented with 10% KH_2_PO_4_ was added weekly for 5 weeks. Soil moisture was maintained at 70% of the field capacity through daily supplementation with distilled water.

**TABLE 1:** The treatments and labels.

Labels	Treatments	Labels	Treatments
L	Soil planted with only *S. nigrum*	LN	Soil planted with *S. nigrum* added with citric acid
LI	Soil planted with *S. nigrum* inoculated with *R. intraradices*	LNI	Soil planted with *S. nigrum* added with citric acid and inoculated with *R. intraradices*

### 2.3 Sample preparation

Soil samples were collected using a quincunx sampling pattern from each pot, sieved through a 100-mesh sieve, and stored at 4°C before analysis. Plants were harvested after 35 days of growth and separated into shoots and roots. The roots were carefully washed with tap water to remove adhering soil particles. Both shoot and root samples were then rinsed thoroughly with distilled water, blotted dry with tissue paper, and weighed. A portion of the fresh root sub-sample from each treatment was used to determine AM colonization. The remaining samples were freeze-dried and stored at 4°C prior to analysis.

### 2.4 Chemical extraction and analytical methods

#### 2.4.1 AM colonization and cadmium quantification

AM colonization was determined using the method described in our previous study (Chen et al., 2017). Dried plant materials were weighed, finely ground, and digested with a concentrated acid mixture of HNO_3_ and HClO_4_ (4:1, v/v). The digest was diluted with distilled water to a final volume of 25 mL. Cadmium (Cd) concentrations in the digest were analyzed using an atomic absorption spectrophotometer (AA-770, Shimadzu).

#### 2.4.2 PBDE extraction and cleanup

Soil samples (1 g) and plant samples (0.1 g) were extracted with 10 mL of a dichloromethane/n-hexane mixture (1:1, v/v) using ultrasonic assistance for 20 min. MBDE-47 and MBDE-209 were added as surrogate standards before extraction. After extraction, the mixtures were centrifuged at 6000 rpm for 2 min, and the supernatants were transferred to heart-shaped bottles. The extracts (approximately 30 mL) were concentrated to ∼2 mL using rotary evaporation, and 10 mL of n-hexane was used to replace the original extracting solution to elute interfering compounds. The eluates were then concentrated again to ∼2 mL via rotary evaporation.

#### 2.4.3 Cadmium speciation in soil and plant tissues

Based on the method of Quevauviller et al. (1993), the chemical forms of Cd in soil were categorized into four fractions: acid-soluble Cd (e.g., carbonate-bound Cd), reducible Cd (e.g., iron and manganese oxide-bound Cd), oxidizable Cd (e.g., organic matter-bound Cd), and residual Cd. In this study, the chemical forms of Cd in *S. nigrum* were determined using a sequential extraction process with specific solutions in the following order: 80% ethanol to extract inorganic Cd (nitrate/nitrite, chloride, and aminophenol Cd); deionized water for water-soluble Cd-organic acid complexes and Cd(H_2_PO_4_)_2_; 1 M NaCl for Cd bound to pectates and protein complexes; 2% acetic acid for insoluble cadmium phosphates (CdHPO_4_, Cd_3_(PO_4_)_2_) and other Cd–phosphate complexes; and 0.6 M HCl to extract cadmium oxalate. This sequential approach allowed for a comprehensive analysis of Cd speciation in *S. nigrum* (Fu et al., 2011).

#### 2.4.4 Column chromatography cleanup procedure

The concentrated extracts were subjected to cleanup and fractionation through a multi-layer chromatographic column (1.5 cm i.d. × 30 cm length) packed following the protocol established by (Li et al., 2015). PBDEs were collected in the second fraction by eluting with 70 mL of dichloromethane/n-hexane mixture (1:1, v/v). The eluate was concentrated to complete dryness under a gentle nitrogen stream and subsequently reconstituted in 2 mL of n-hexane. All processed samples were stored at 4°C in amber vials until analysis. Immediately prior to GC-MS analysis, 50 μL of FBDE-4003S (100 ng/μL in n-hexane) was added as an internal standard for quality control purposes.

#### 2.4.5 Instrumental analysis of PBDEs

The analysis of PBDEs was performed using an Agilent 7890A gas chromatograph (Agilent Technologies, United States) coupled with a 5975C mass selective detector (MSD). The system was configured with a DB-XLB fused silica capillary column (15 m × 0.25 mm i.d. × 0.1 μm film thickness) and operated in negative chemical ionization (NCI) mode. The GC oven temperature program was initiated at 80°C (1 min hold), ramped at 10°C/min to 200°C, then increased at 20°C/min to 300°C (15 min final hold). High-purity helium (99.999%) was used as carrier gas at a constant flow rate of 1.5 mL/min. The injection port was maintained at 280°C, while the ion source, transfer line, and quadrupole temperatures were set at 230°C, 290°C (Aux-2), and 150°C, respectively.

### 2.5 Bioconcentration factor analysis of Cd and BDE-209

The differences in the capacity of *S. nigrum* to accumulate Cd and BDE-209 under different treatments were determined based on the bioconcentration factor (BF), which is defined in the equation:


$$\text{BF}=\frac{\text{C}\,d\,\mathrm{or}\,\mathrm{BDE}-209\,\mathrm{concentration}\,\mathrm{in}\,\mathrm{shoots}}{\text{C}\,d\,\mathrm{or}\,\mathrm{BDE}-209\,\mathrm{concentration}\,\mathrm{in}\,\mathrm{soil}}$$

### 2.6 Enzymatic activity assay

A mixture of 1.0 g soil and 10 mL of 1% pyrogallic acid was incubated at 30°C for 2 h, after which 4 mL of citric-phosphoric acid buffer (pH = 4.5) was added. The purpurogallin formed was extracted with chloroform and measured spectrophotometrically at 430 nm. The results are expressed as mg PPG g^–1^ dry soil per 2 h (Dick et al., 1988). Dehydrogenase activity was quantified through TTC reduction to TPF, with results expressed as mg TPF kg^–1^ dry soil day^–1^ (Chen et al., 2010). Urease activity was assessed via phenol-hypochlorite reaction, measuring NH_3_-N production per 5.0 g soil (Sumner and Somers, 2014).

### 2.7 Quality assurance and quality control

A procedure blank was included in each batch of extractions to monitor potential contamination. Recovery tests for PBDEs were conducted by spiking soil and plant samples with known concentrations of MBDE-47 and MBDE-209. Recoveries ranged from 84 to 102% for soil samples and 85% to 110% for plant samples. For Cd, a certified standard material was digested alongside the plant samples to verify the accuracy of the digestion procedure, achieving a recovery rate of 90%.

### 2.8 Data analysis

All data were processed using SPSS 16.0 (IBM, United States) for statistical computations, with results presented as mean ± standard deviation (*n* = 3 biological replicates). Significant differences among treatments were evaluated by one-way ANOVA, with post-hoc Duncan’s test (*p* < 0.05) for multiple comparisons. Graphical representations were created using Origin 2024 (OriginLab, United States).

## 3 Results

### 3.1 Colonization rates and biomass

**FIGURE 1:**
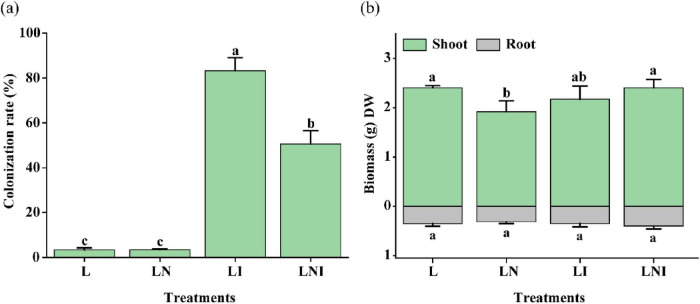
Colonization rates **(a)** and dry biomass **(b)** of *S. nigrum* under CA and AM fungi (mean ± S.D., *n* = 3). The four treatments were L (Ctrl), LN (+CA), LI (+AM fungus), and LNI (+AM fungus +CA). “DW” represents dry weight. The same lowercase letters indicate no significant differences among treatments (*p* < 0.05). ANOVA followed by Duncan’s test was used to assess the significance of differences among groups. The same applies to the following analyses.

The addition of CA reduced colonization rates by 39.23%, from 83.19% in LNI to 50.55% in LI, suggesting CA inhibited AM fungal colonization (Figure 1a). Although CA is widely recognized as an effective organic chelating agent for enhancing the phytoextraction of soil pollutants, its single application in this study reduced the biomass of *S. nigrum* by 20.22%. In contrast, the inoculation of AM fungi (LI) did not significantly increase plant biomass. Notably, the combinatory application of AM fungi and CA (LNI) effectively mitigated the negative impact of CA (LN). Specifically, shoot biomass in LNI increased by 25.30% compared to LN. Root biomass, however, remained consistent across all treatments.

### 3.2 Uptake of Cd in *S. nigrum*

**FIGURE 2:**
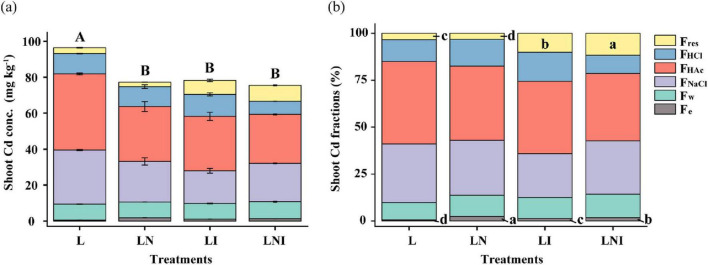
Concentration **(a)** and proportions **(b)** of different fractions of Cd in *S. nigrum*. The same capital letters indicate no significant differences in total Cd concentration among treatments, while the same lowercase letters indicate no significant differences in the proportion of different Cd fractions among treatments (*p* < 0.05).

Compared to L treatment, both CA and AM fungi reduced Cd concentrations in the shoot (Figure 2a). Further analysis of Cd fractions in the shoot revealed that the proportion of F_*e*_-Cd (extracted with 80% ethanol) in LN was 42.64%–281.20% higher than that in the other treatments (Figure 2b). In contrast, AM fungal inoculation (LI and LNI) reduced the amount of F_*e*_-Cd while increasing the proportion of F_*res*_-Cd. The F_*res*_-Cd proportion in LNI was 12.39% higher than that in LN. Notably, inorganic Cd forms, such as F_*e*_-Cd, exhibited higher mobility and posed greater cytotoxicity to plant cells. In contrast, F_*res*_-Cd, commonly regarded as residual Cd bound to stable organic or inorganic matrices within the plant, demonstrated the lowest bioavailability and caused minimal damage to plants (Li et al., 2018b).

### 3.3 Cd concentration and fraction in soil

**FIGURE 3:**
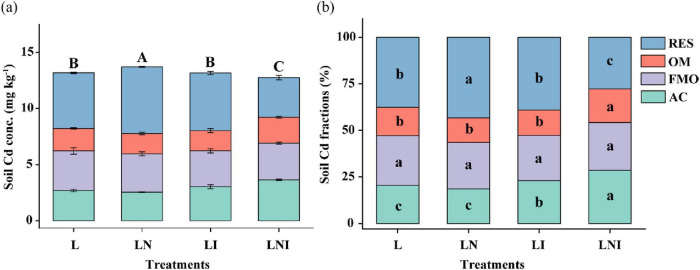
Concentration **(a)** and proportion **(b)** of different fractions of Cd in soil. The same capital letters indicate no significant differences in total Cd concentration among treatments, while the same lowercase letters indicate no significant differences in the proportion of different Cd fractions among treatments (*p* < 0.05).

As shown in Figure 3, the Cd concentration and fractions have changed in the rhizosphere soil due to CA and AM fungi. Compared to L treatment, CA increased the total Cd concentration in the soil, with the proportion of residual Cd (RES-Cd) increasing by 19.66%. While single AM fungi did not affect the soil Cd concentration, the acid-soluble Cd (AC-Cd) increased by 12.29%. The combined application of AM fungi and CA (LNI) reduced the Cd concentration by 13.09 and 10.18% compared to L and LI treatments, respectively. Furthermore, LNI treatment exhibited the highest proportion of AC-Cd and the lowest proportion of RES-Cd. These findings suggest that the combined application of AM fungi and CA facilitates the transformation of residual Cd (mineral-bound and less bioavailable forms) into more bioavailable and plant-accessible Cd fractions, such as acid-soluble Cd.

### 3.4 Uptake and translocation of PBDEs in *S. nigrum*

**FIGURE 4:**
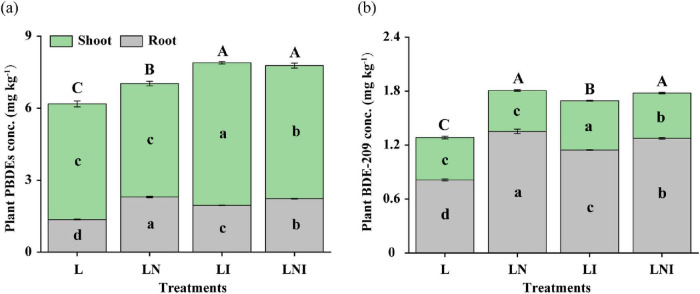
PBDEs **(a)** and BDE-209 **(b)** concentration in shoots and roots. The same capital letters indicate no significant differences in total concentration of PBDEs or BDE-209 in plants, while the same lowercase letters indicate no significant differences in the concentration of PBDEs or BDE209 in organs (shoot and root) among different treatment (*p* < 0.05).

As shown in Figure 4a, single inoculation with CA (LN) or AM fungi (LI) significantly increased plant PBDEs concentrations compared to the control (L). The highest shoot PBDEs concentration was observed in LI treatment, which increased by 83.33 and 22.22% compared to L and LN treatments, respectively. The LNI treatment ranked second, with shoot PBDEs concentrations increasing by 61.11 and 5.56% compared to L and LN treatments, respectively, suggesting that the combined treatment promotes PBDEs translocation to shoots. For roots, CA inoculation (LN) doubled the PBDEs concentration compared to the control, while LNI treatment reduced root PBDEs accumulation by 16.67% compared to LN but increased shoot concentrations by 5.56%.

The trends for BDE-209 concentrations were similar (Figure 4b). Single inoculation with CA (LN) or AM fungi (LI) enhanced BDE-209 accumulation compared to the control, while the combined treatment (LNI) further increased concentrations compared to LI. The highest shoot BDE-209 concentration occurred under LI treatment, with increases of 41.67 and 25.00% compared to L and LN treatments, respectively. LNI treatment also significantly elevated shoot BDE-209 concentrations, showing increases of 31.25 and 10.93% compared to L and LN, respectively, further highlighting the role of AM fungi in promoting shoot accumulation of BDE-209.

In the roots, CA inoculation (LN) had the strongest effect, increasing root BDE-209 concentration by 66.34% compared to L. However, LNI treatment slightly reduced root BDE-209 concentrations by 5.77% compared to LN, while increasing shoot concentrations by 10.93%, reinforcing its role in facilitating BDE-209 translocation to shoots. Additionally, AM fungi may have contributed to the degradation and debromination of BDE-209, as evidenced by lower overall BDE-209 concentrations in LI treatment compared to LN.

### 3.5 Debromination and dissipation of BDE-209 in soil

**FIGURE 5:**
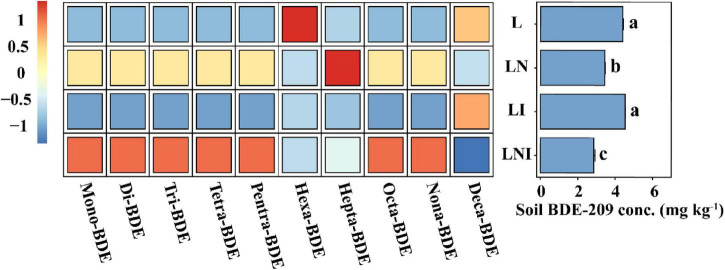
Concentrations of BDE-209 residues and its debromination products in soil. **(Left)** Concentrations of BDE-209 debromination products, with data min - max normalized to [–1, 1]. **(Right)** Residual BDE-209 concentrations in soil. The same lowercase letters indicate no significant differences among treatments (*p* < 0.05).

Soil BDE-209 can be absorbed by plants while simultaneously undergoing debromination and dissipation driven by rhizosphere microbial activity (Huang et al., 2010). To further elucidate the contribution of CA and AM fungi to BDE-209 dissipation via their influence on microbial activity, we analyzed the concentration of residual BDE-209 in the soil along with the levels of its dibrominated products (Figure 5). The BDE-209 content in soil exhibited significant differences among the treatments. The application of CA reduced the BDE-209 content in soil, with LN treatment achieving a reduction of 21.68%. When combined with AM fungi, the degradation rate was further enhanced to 35.21%, likely due to the synergistic effects of CA and AM fungi in enhancing microbial activity and enzyme functions. However, the application of AM fungi alone did not significantly affect the BDE-209 content. By analyzing the debromination products of BDE-209 in soil, it was observed that LN and LNI treatments accelerated the debromination process, promoting the transformation of BDE-209 into lower-brominated forms.

### 3.6 Soil enzyme activities

**FIGURE 6:**
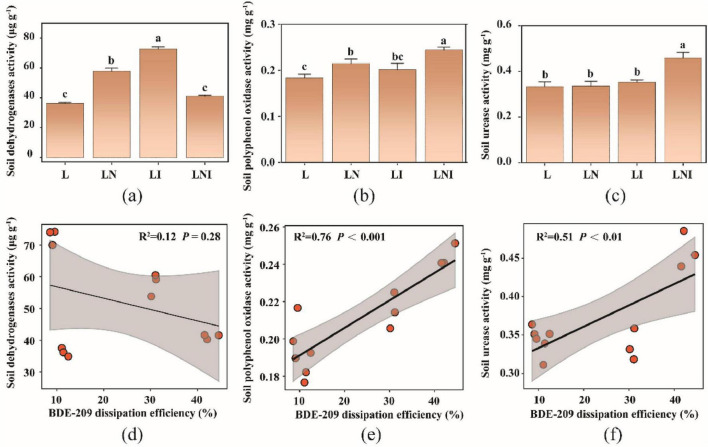
Soil enzyme activities **(a–c)** and the spearman correlation with BDE-209 dissipation efficiency **(d–f)**. The same lowercase letters indicate no significant differences among treatments (*p* < 0.05).

Our previous research demonstrated a correlation between soil enzyme activity and BDE-209 degradation. Therefore, we measured the activities of dehydrogenase, polyphenol oxidase, and urease, which are involved in microbial metabolism and soil redox processes (Li et al., 2020). Figure 6a shows that soil dehydrogenase activity was significantly higher under the LI treatment compared to other treatments, whereas the LNI treatment exhibited a moderate increase. Figure 6b indicates that polyphenol oxidase activity was significantly enhanced in LNI treatment, followed by LN treatment, while no significant difference was observed between LN and L treatments. Figure 6c reveals that urease activity was also significantly elevated in LNI treatment compared to other treatments.

Figures 6d–f further analyzes the correlation between enzyme activities and BDE-209 dissipation efficiency. A strong positive correlation was observed between BDE-209 dissipation efficiency and polyphenol oxidase activity (*R*^2^ = 0.76, *P* < 0.001) and urease activity (*R*^2^ = 0.51, *P* < 0.01), indicating their critical role in enhancing BDE-209 degradation. In contrast, no significant correlation was found between dehydrogenase activity and BDE-209 dissipation efficiency (*R*^2^ = 0.12, *P* = 0.28), suggesting that this enzyme is less involved in the degradation process.

## 4 Discussion

### 4.1 Impact of citric acid and AM fungi on Cd biomass in co-contaminated Soil

Citric acid (CA) has been widely recognized for its ability to enhance the dissolution and mobilization of metal elements in soil, and it is commonly used as a chelating agent to facilitate the phytoremediation of Cd-contaminated soils by *S. nigrum* (Sun et al., 2006). However, in this study, CA significantly reduced the biomass of *S. nigrum* growing in soil co-contaminated with Cd and BDE-209 (Figure 1b). This finding aligns with previous studies demonstrating that 5 mM CA decreased the shoot biomass of *Juncus effusus* by increasing Cd availability and uptake, ultimately exerting a negative impact on plant growth (Chen et al., 2003). In the present study, under the same CA treatment, the shoot Cd concentration in LN treatment was reduced by 19.91% compared to L treatment, while the proportion of ethanol-extractable Fe-Cd increased by 2.81-fold. Fe-Cd, a more chemically active form of Cd, readily binds to critical intracellular molecules such as proteins and DNA, exacerbating oxidative stress and cellular damage (Yu et al., 2024). This increased proportion of Fe-Cd may be a key factor contributing to the reduction in *S. nigrum* biomass in CA-treated plants.

According to a previous study that summarized global data, the plants inoculated with AM fungi showed colonization rates exceeding 50% (Figure 1a), indicating successful fungal colonization (Ma et al., 2023). The LNI treatment reduced AM fungal colonization by 39.23% compared to LI, consistent with Qiu et al. (2021). Although AM fungi can tolerate acidic conditions (Hu et al., 2022), excessive acidification can still inhibit their growth and colonization. The initial soil pH of 5.9 was further lowered by the addition of 5 mM citric acid, which likely contributed to the observed decline in colonization. This is consistent with previous reports that AM fungal biomass and diversity significantly decrease when soil pH drops below critical thresholds (pH = ∼6.5) due to nitrogen-induced acidification (Wu et al., 2023). In both LI and LNI treatments, the proportion of Fe-Cd was significantly higher than in the L treatment. However, no significant differences in biomass were observed between these treatments and L treatment. This could be attributed to the significantly higher proportion of Fres-Cd (residual Cd) in the LI and LNI treatments. Fres-Cd primarily exists as Cd bound to the cell wall or precipitated as insoluble compounds, making it metabolically inactive and thus less toxic to plants (Guo et al., 2024). Furthermore, the growth-promoting effects of AM fungi likely counteracted the negative impacts associated with the increased Fe-Cd proportion (Wang et al., 2025).

### 4.2 Impact of CA and AM fungi on soil Cd activation and plant uptake

The concentrations of Cd in shoots of *S. nigrum* were significantly reduced under the treatment of CA, AM fungi, or their combination, compared with the control (L) (Figure 2). Similarly, it has been reported that the addition of 3 g kg^–1^ CA caused a significant reduction in Cu concentration in shoots and roots of *Z. mays* in Cu and pyrene co-contaminated soil (Chigbo and Batty, 2015). In the present study, the significant decrease in Cd concentrations under CA treatment could be attributed to the lower stability of the formed Cd complexes. Additionally, the biodegradation of CA in soil may increase soil pH due to the consumption of H^+^ from carboxylic acid and the release of OH^–^ and CO_2_. This would further lead to a lack of complexing agents, consequently reducing the bioavailability of Cd to *S. nigrum* (Chen et al., 2003). Therefore, the concentration of Cd in *S. nigrum* declined accordingly. Single CA application (LN) significantly reduced Cd concentration and biomass in the shoots of *S. nigrum*, resulting in Cd concentration in the rhizosphere soil being significantly higher than that in the control (L) (Figure 3).

Contrary to our results, Jiang et al. (2016) found that *Funneliformis mosseae* significantly enhanced Cd uptake by *S. nigrum* in single Cd-contaminated soil. These contrasting results could be attributed to the functional diversity of different AM fungi strains (Li et al., 2011). Liu et al. (2011) also reported that the inoculation of *Rhizophagus intraradices*, *Glomus constrictum*, or *Funneliformis mosseae* significantly reduced Cd concentrations in the shoots and roots of marigold plants in soil amended with 5 or 50 mg kg^−1^ Cd. In our study, Cd uptake by *S. nigrum* was reduced (Figure 2, LI), which might be attributed to the functional diversity of different AM fungi strains and their varying effects on metal uptake. This discrepancy in results could also be explained by differences in soil conditions, plant species, or inoculum concentrations used in the studies. The exudates, such as glomalin, a glycoprotein produced by AM fungi, can strongly and irreversibly sequester Cd in the rhizosphere, potentially limiting Cd availability for plant uptake (Chen et al., 2001).

The combined treatment of CA and AM fungi (LNI) significantly decreased the Cd concentration in the soil. Our results showed that the concentration of acid-soluble Cd in soils treated with AM fungi increased, and this concentration was further enhanced when CA was used in combination with AM fungi. This suggests that the combined application of CA and AM fungi (LNI) promotes the activation of Cd in the soil, thereby facilitating Cd uptake by plant roots. Although there was no significant increase in Cd concentration in the shoots of the plants, AM fungi hyphal concentrations in the soil exceeded 2.5 m g^–1^ soil (Xiang et al., 2014). The exudates produced by AM fungi may interact with Cd and other heavy metals, such as Cr, forming insoluble complexes or phosphate-like substances that are immobilized within the fungal structure (Wu et al., 2015). Additionally, AM fungi hyphae are capable of adsorbing nanoplastics, which are sequestered within the fungal structure, reducing the uptake of nanoplastics by the roots and their subsequent translocation to the shoots (Li H. H. et al., 2024). As a result, a significant portion of Cd may be immobilized in the AM fungi structure without being translocated to the shoots of the plants, leading to a reduction in Cd concentration in the soil. This resulted in a decrease in the bioconcentration factor under the LNI treatment, while the removal efficiency of Cd in the soil was improved (Table 2).

**TABLE 2:** Bioconcentration factors and removal efficiencies of Cd and BDE-209 by *S. nigrum*.

Treatments	Cd		BDE209	
	**Bioconcentration factor (BF)**	**Removal efficiency (%)**	**Bioconcentration factor (BF)**	**Dissipation efficiency (%)**
L	7.53 ± 0.58a	10.62 ± 0.65b	1.07 ± 0.11d	11.6 ± 0.74c
LN	6.14 ± 0.55b	7.35 ± 0.50c	1.32 ± 0.15b	30.8 ± 2.11b
LI	6.36 ± 0.50b	10.48 ± 0.60b	1.21 ± 0.12c	9.06 ± 0.52c
LNI	6.07 ± 0.59b	12.64 ± 0.65a	1.77 ± 0.10a	42.8 ± 3.10a

The same lowercase letters indicate no significant difference between treatments (*P* < 0.05).

### 4.3 Synergistic effects of citric acid and AM fungi on BDE-209 dissipation and microbial enzyme activity enhancement in soil

LN, LI, and LNI treatments had a negative effect on Cd uptake but a positive influence on BDE-209 uptake, implying that there might be competition between Cd and BDE-209 uptake by *S. nigrum* (Pan et al., 2024). The addition of CA, AM fungi, or their combination all elevated the BDE-209 concentrations in shoots and roots (Figure 4). Citric acid might act as a surfactant, reducing the sorption of BDE-209 on soil particles and thereby increasing its bioavailability and improving plant uptake (Gong et al., 2024). Currently, only one study has investigated the effects of AM fungi on BDE-209 uptake by plants, reporting that BDE-209 concentrations in ryegrass roots were significantly increased with the inoculation of *Funneliformis mosseae* (Wang et al., 2011). The colonization by AM fungi resulted in the formation of extraradical hyphae, which can access sites, such as fine soil pores, that are unavailable to plant roots, and transfer pollutants from soil to plants (Gao et al., 2010a). BDE-209 is generally considered to have low toxicity, but increasing evidence shows that it can be decomposed into other prohibited PBDEs with much higher toxicity. Therefore, it is important to investigate the uptake and subsequent debromination of BDE-209 in *S. nigrum* (Ma et al., 2024). Overall, all treatments significantly increased the total concentration of PBDEs in the roots, and treatments inoculated with AM fungi increased the concentration in the shoots, which is consistent with the results of BDE-209 concentration in *S. nigrum* (Figure 4). Furthermore, the total concentrations of the debrominated metabolites were higher in shoots than in roots, indicating that BDE-209 might be first absorbed by roots, then translocated to shoots, and finally degraded into lower brominated products in shoots (Wang et al., 2011). Meanwhile, The total PBDEs in the LI treatment were significantly higher than those in the LN treatment, but the total amount of BDE-209 showed the opposite trend. This might be because BDE-209 was degraded into lower brominated products after being translocated to the shoots. In plants, PBDEs undergo debromination, hydroxylation, and methoxylation reactions (Dobslaw et al., 2021). However, the absorption and transformation of PBDEs by plants largely depend on the microbial community already established within the plant. The inoculation of AM fungi alters the structure of the plant’s endophytic bacterial community, which may contribute to enhanced degradation of PBDEs. This process could also be influenced by the electron transfer generated during plant physiological processes, which facilitates the breakdown of PBDEs (Wang Y. et al., 2022).

The observed decrease in soil BDE-209 concentrations likely reflects multiple dissipation pathways, including microbial degradation and phytoextraction. Given its high hydrophobicity (log KOW ≈ 10) and negligible vapor pressure, volatilization losses were considered insignificant in this system (Lu and Zhang, 2014). The significant reduction in soil BDE-209 concentration across all treatments suggests that both CA and AM fungi promoted its dissipation. The highest dissipation efficiency (42.8%) was observed in the LNI treatment, followed by LN (30.8%), indicating that the combination of CA and AM fungi had a synergistic effect. Given the negligible contribution of plant uptake and volatilization to BDE-209 loss, microbial activity is likely the primary driver of its degradation in soil. The changes in enzymatic activities further support this hypothesis. Among the enzymes analyzed, polyphenol oxidase activity showed the strongest correlation with BDE-209 dissipation efficiency (*R*^2^ = 0.76, *P* < 0.001), suggesting a potential role in its oxidative degradation. The LNI treatment exhibited the highest polyphenol oxidase activity, consistent with its dissipation efficiency. This suggests that CA and AM fungi enhanced microbial activity and increased the abundance of oxidative enzymes, thereby accelerating the breakdown of BDE-209 (Li et al., 2018c). While this correlation highlights a potential mechanistic link, other oxidoreductase enzymes or microbial community dynamics may also contribute to BDE-209 degradation, and causal relationships require further validation.

## 5 Conclusion

In this study, the synergistic effects of citric acid (CA), arbuscular mycorrhizal (AM) fungi, and their combination were investigated for enhancing the phytoremediation of Cd and BDE-209 co-contaminated soil using the hyperaccumulator *S. nigrum*. The addition of CA significantly increased ethanol-extractable Cd in shoots by 2.81-fold but reduced shoot biomass due to elevated Cd toxicity. In contrast, AM fungi increased the proportion of residual Cd in shoots, thereby reducing Cd toxicity to plants, and enhanced the proportion of acid-soluble Cd in soil, promoting Cd mobilization, but single AM fungi did not significantly alter the remaining Cd concentration in the soil. Notably, the combined treatment of CA and AM fungi (LNI treatment) reduced soil Cd concentration by 13.09% compared to the control, demonstrating their synergistic potential for Cd immobilization and mobilization. Additionally, a significant positive correlation was observed between soil polyphenol oxidase activity, urease activity, and BDE-209 dissipation efficiency. The LNI treatment achieved the highest BDE-209 dissipation efficiency (42.8%), attributed to the enhanced microbial activity and enzymatic degradation facilitated by the combined application of CA and AM fungi. These findings highlight that the integration of *S. nigrum*, CA, and AM fungi offers a promising strategy for the remediation of Cd and BDE-209 co-contaminated soils. However, the potential environmental risks associated with the degradation products of BDE-209 warrant further investigation.

## Data Availability

The raw data supporting the conclusions of this article will be made available by the authors, without undue reservation.
